# C-4 Gem-Dimethylated Oleanes of *Gymnema sylvestre* and Their Pharmacological Activities

**DOI:** 10.3390/molecules181214892

**Published:** 2013-12-04

**Authors:** Giovanni Di Fabio, Valeria Romanucci, Mauro Zarrelli, Michele Giordano, Armando Zarrelli

**Affiliations:** 1 Department of Chemical Sciences, University Federico II, Complesso Universitario Monte S. Angelo, Via Cintia 4, Napoli 80126, Italy; E-Mails: difabio@unina.it (G.D.F.); valeria.romanucci@unina.it (V.R.); 2 IMCB-Institute of Composite and Biomedical Materials CNR–National Research Council P E Fermi, (Granatello) Portici, Napoli 80055, Italy; E-Mails: mauro.zarrelli@imcb.cnr.it (M.Z.); michele.girdano@imcb.cnr.it (M.G.)

**Keywords:** *Gymnema sylvestre*, triterpenoids, oleanes, pharmacological activities, phytochemistry

## Abstract

*Gymnema sylvestre* R. Br., one of the most important medicinal plants of the Asclepiadaceae family, is a herb distributed throughout the World, predominantly in tropical countries. The plant, widely used for the treatment of diabetes and as a diuretic in Indian proprietary medicines, possesses beneficial digestive, anti-inflammatory, hypoglycemic and anti-helmentic effects. Furthermore, it is believed to be useful in the treatment of dyspepsia, constipation, jaundice, hemorrhoids, cardiopathy, asthma, bronchitis and leucoderma. A literature survey revealed that some other notable pharmacological activities of the plant such as anti-obesity, hypolipidemic, antimicrobial, free radical scavenging and anti-inflammatory properties have been proven too. This paper aims to summarize the chemical and pharmacological reports on a large group of C-4 gem-dimethylated pentacyclic triterpenoids from *Gymnema sylvestre*.

## 1. Introduction

Plant natural products from abundant species represent attractive and sustainable starting materials for the preparation of new bioactive substances. *Gymnema sylvestre* R. Br. is a valuable herb from the family Asclepiadaceae, widely distributed in India, Malaysia, Sri Lanka, Australia, Indonesia, Japan, Vietnam, tropical Africa and Southwestern China. Other scientific and common names in different languages are given in Table 1.

**Table 1:** Scientific and common names of *Gymnema sylvestre* [1,2,3].

	Scientific names
	*Gymnema sylvestre*, *Asclepias geminata*, *Asclepias geminata*, *Periploca sylvestris*, *Gymnema melicida*
**Language**	**Common names**
**English**	*Gymnema*, *Cowplant*, *Australian cowplant*, *Gurmari*, *Gurmarbooti*, *Gurmar*, *Periploca of the woods*, *Meshasringa*, *Gemnema Melicida*, *Gimnema*, *Gur-Mar*, *Gymnema montanum*, *Gymnéma*, *Gymnéma Sylvestre*, *Miracle plant*, *Periploca sylvestris*, *Shardunika*, *Vishani*, *Ram’s horn*, *Miracle fruit*, *Merasingi*, *Small Indian ipecac*, *Sugar destroyer*
**Sanskrit**	*Meshashringi*, *Madhunashini*, *Ajaballi*, *Ajagandini*, *Bahalchakshu*, *Karnika*, *Chakshurabahala*, *Kshinavartta*
**Marathi**	*Kavali*, *Kalikardori*, *Vakundi*
**Hindi**	*Gurmar*, *Merasingi*
**Marathi**	*Kavali*, *Kalikardori*, *Vakundi*
**Gujrathi**	*Dhuleti*, *Mardashingi*
**Telugu**	*Podapatri*
**Tamil**	*Adigam*, *Cherukurinja*, *Sarkarikolli*
**Kannada**	*Sannager-asehambu*
**Malayalam**	*Chakkarakolli*, *Madhunashini*
**Bengali**	*Mera-Singi*

*Gymnema sylvestre* (*GS*) is considered to have potent anti-diabetic properties. This plant is also used for controlling obesity, in the form of Gymnema tea, and it is often called “gurmar” (destroyer of sugar), as chewing the leaves causes the loss of the ability to taste sweetness [4,5]. Extracts of its leaves and roots are used in India and parts of Asia as a natural treatment for diabetes, as they help lower and balance blood sugar levels [6]. In addition, the plant possesses antimicrobial [7], anti-hyphal [8] anti-hypercholesterolemic [9], and hepatoprotective activities [10]. It also acts as a feeding deterrent to the caterpillar *Prodenia eridania* [11], prevents dental caries caused by *Streptococcus mutans* [12] and is used in cosmetics [13]. In addition, it is also used in the treatment of rheumatism, cough, ulcer, jaundice, dyspepsia, constipation, asthma, eye complaints, inflammations and snake bites [14,15]. The taxonomy of the plant is described in Table 2 [16].

**Figure 1:**
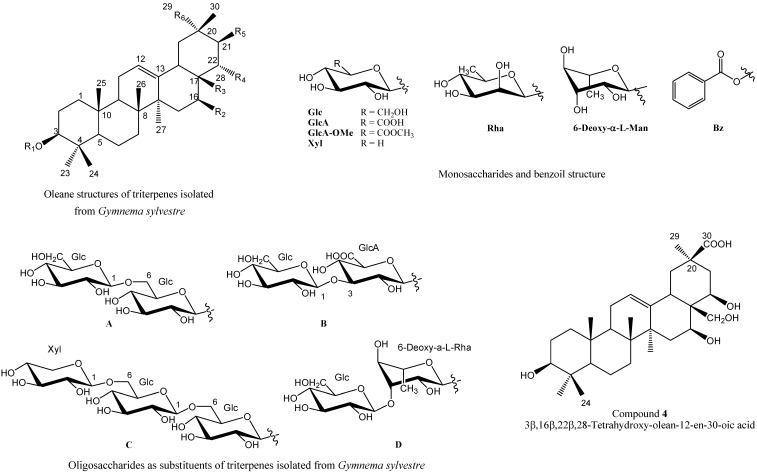
General structures of oleane triterpenes isolated from *Gymnema sylvestre*.

This paper aims to summarise the chemical and pharmacological reports on oleanes, a large group of pentacyclic triterpenoids gem-dimethylated at C-4 (Figure 1). The oleane skeleton includes a C12:13 double bond. The most common functional group in this class is the 3-OH substituent, which is present in the precursor squalene. Hydroxyl groups, free or acylated, are also present at C-16, C-21, C-22, C-28, and C-30. The first section of this review provides a general description of the plant, its chemical composition, extraction methods and uses. Then, the IUPAC names, biological activities and physical and chemical characterisation (IR, ^1^H and ^13^C-NMR, [α]_D_, X-ray, MS, *etc*.) of these compounds are described. These data have been obtained from various tables and references.

**Table 2:** Taxonomy of *Gymnema sylvestre*.

Kingdom	Plantae
Subkingdom	Tracheobionta
Superdivision	Spermatophyta
Division	Magnoliophyta
Class	Magnoliopsida
Subclass	Asteridae
Order	Gentianales
Family	Asclepiadaceae
Genus	*Gymnema* R. Br.
Species	*sylvestre*

## 2. Plant Description

*GS* is a vulnerable species. It is a slow growing large perennial and medicinal woody climber distributed throughout India in dry forests up to 600 m in height. *GS* is distributed across a large area, occurring from East Africa to Saudi Arabia, India, Sri Lanka, Vietnam and southern China, as well as Japan (Ryukyu Islands), the Philippines, Malaysia, Indonesia and Australia. In addition, it occurs throughout most of West Africa and extends to Ethiopia and South Africa [16,17]. The leaves of *GS* are green; the stems are hairy and light brown. The leaves are 2–6 cm in length and 1–4 cm in width and are simple, petiolate and opposite, with an acute apex and reticulate venation. They are pubescent on both surfaces [1,18]. They have a characteristic odour; the taste is slightly bitter and astringent. The leaves also possess the remarkable ability to inhibit the perception of sweet tastes for a few hours [19,20]. The flowers are small, yellow, in axillary and lateral umbel like cymes. The lobes of the calyces are long, ovate, obtuse and pubescent. The corolla is pale yellow, campanulate and valvate with a single corona and five fleshy scales [16,21,22,23]. The powdered material is slightly yellowish-green and is bitter tasting with a pleasant aromatic odour. When the powder is treated sequentially with 1 M aqueous NaOH and 50% KOH, it exhibits green fluorescence under UV at 254 nm; with 50% HNO_3_ in daylight, an orange colour is observed. The dilute hydro-alcoholic extract suppresses the ability to taste sweetness, foams copiously when shaken with water and forms a voluminous precipitate upon addition of dilute acid [22,24,25,26].

## 3. Chemical Composition

*GS* contains triterpene saponins belonging to the oleane and dammarene classes. Other constituents include formic, butyric and tartaric acids, flavones, anthraquinones, hentriacontane, pentatriacontane, α- and β-chlorophylls, phytin, resins, δ-quercitol, lupeol, β-amyrin-related glycosides and stigmasterol [27]. The plant extract also tests positive for alkaloids. In addition, the leaves yield acidic glycosides and anthraquinone derivatives. The pharmacological activities of *GS* are listed below.

## 4. Pharmacological Studies

### 4.1. Anti-Diabetic Activity and Mechanism of Action

*GS* has shown promising results as an anti-diabetic agent. The first scientific confirmation of the efficacy of *GS* in human diabetes came almost a century ago, when it was demonstrated that *GS* leaves reduce urinary glucose levels in diabetic subjects [28]. No adverse effect was observed; thus, it can be concluded that the powdered extract lowers fasting and postprandial blood glucose levels [2].

Several studies suggest that the hypoglycaemic activity of *GS* is due to stimulation of insulin release (and possibly regeneration of Langerhans islet β-cells), modulation of the enzymes responsible for glucose utilisation (increased phosphorylase activity and the decreased activity of gluconeogenic enzymes and sorbitol dehydrogenase) and inhibition of glucose absorption in the bowel [29,30,31,32]. Numerous studies using animal models have confirmed the hypoglycaemic effect of *GS* [33,34]. Microscopy of a group of animals receiving *GS* extract showed that the nuclei of β-endocrinocytes were significantly enlarged in all sections of the pancreas. Pancreatic islets occupied the same volume fraction and area [35], supporting the hypothesis that the use of *GS* increases the endogenous levels of insulin, possibly due to regeneration of pancreatic β-cells [36]. Other effects of *GS* extract include a prolonged hypoglyacemic action of exogenous insulin in dogs without a pancreas, an intensification of the effects of insulin, and an extended duration of reduced glucose levels [29]. Use of *GS* extracts to treat diabetes in rats significantly increases life expectancy. Plasma insulin was increased in mice with streptozotocin-induced diabetes mellitus (DM) upon administration of the saponin fraction of the methanol extract of *GS* leaves or isolated triterpene glycosides [9]. The use of *GS* extracts for 21 days after streptozotocin intoxication reliably reduced plasma glucose levels, increased insulin levels (most likely due to increased membrane permeability rather than stimulation of exocytosis by other pathways) [37], and normalised high-density lipoprotein (HDL) [35] concentrations. Glycaemia and insulinaemia were restored to normal over 60 days in rats with diabetes after peroral administration of the alcohol fraction of *GS* extract. The number of Langerhans islets and β-cells in the pancreas doubled [31,38]. In 1990, the effects of a *GS* extract on type 1 and 2 DM were published [30]. Clinical trials were conducted in the USA on the patented preparation ProBeta, based on a *GS* extract [30,31,39,40]. Treatment of 27 patients with type 1 DM who were on insulin therapy with *GS* extract (400 mg/d) showed reduced fasting glucose levels, up to 35%, and normalised serum lipid concentrations. The required dose of exogenous insulin was reduced (up to 50%), and the level of endogenous insulin was increased 12 months after the start of treatment. It was proposed that *GS* increased the endogenous levels of insulin and C-peptide, possibly due to pancreatic regeneration.

### 4.2. Inhibitory Effects on Palatal Taste Response

*Gymnema* leaf and root extracts have been shown to affect the palatal taste response by interfering with the ability of the taste buds on the tongue to perceive sweet and bitter tastes. It is believed that inhibiting the perception of sweet taste may cause people taking it to limit their intake of sweet foods. This activity may be partially responsible for its hypoglycaemic effect [41]. The active compounds are thought to act by binding to the sweet taste receptor protein [42].

### 4.3. Hypolipidemic Activity

The altered blood lipid levels and hyperglycaemia associated with DM increase the risk of atherosclerosis [43]. Therefore, it is significant that *GS* not only possesses hypoglycaemic properties but can also correct impaired lipid exchange [9]. Extracts of *GS* have shown anti-atherosclerotic potential, with efficacy similar to the standard lipid-lowering agent clofibrate [9]. *In vivo* studies have been conducted primarily with rats. Significant decreases in fat absorption were observed when *GS* extract was administered orally to rats fed a high-fat or normal-fat diet. The administration of leaf extracts to hypolipidaemic rats for two weeks was found to reduce elevated serum triglycerides, total cholesterol, very low-density lipoprotein and low-density lipoprotein levels in a dose-dependent manner [4,8]. An aqueous extract of *GS* was shown to suppress the accumulation of lipids in the liver of rats on a high fat diet to the same extent as chitosan [44]. Spontaneously hypertensive rats consuming extracts of *GS* showed a decrease in circulating cholesterol concentrations [45].

### 4.4. Anti-Obesity Activity

Obesity is a well-established risk factor for cardiovascular disease, diabetes, hyperlipidaemia, hypertension, osteoarthritis, and stroke. *GS* promotes weight loss through its ability to reduce sweet cravings and to control blood sugar levels [2,3,42,46]. A standardised *GS* extract in combination with niacin-bound chromium and hydroxycitric acid was evaluated for anti-obesity activity by monitoring changes in body weight, body mass index, appetite, lipid profiles and the excretion of urinary fat metabolites in moderately obese human volunteers.

### 4.5. Anti-Cancer Activity

The alcoholic extract of *GS* is a potent anti-cancer agent against the A549 (human lung adenocarcinoma) and MCF7 (human breast carcinoma) cell lines [47]. Additionally, the alcoholic extract of *GS* has been shown to inhibit intestinal breast cancer resistance protein (BCRP) [48]. BCRP inhibition enhances the systemic availability of orally-administered drugs, including topotecan, irinotecan, nitrofurantoin, and sulfasalazine, by increasing absorption [48,49]. For this reason, *GS* extracts possess potent medicinal value in cancer treatment.

### 4.6. Haemolytic Activity and Mechanism of Action

Triterpene saponins usually show high haemolytic activity, whether they are neutral, acidic, or an ester saponin. Polar substituents on ring A and weakly polar substituents on rings D and E increase the lysis of erythrocyte membranes, which results in haemoglobin release. Triterpenes, acylglycosides, and saponins with strongly polar substituents on rings D and E exhibit a lower haemolytic activities [50,51]. With respect to the carbohydrate moiety, the situation is less clear. Haemolysis usually decreases with length and increases with branching of the sugar chain [52].

The molecular mechanism of these effects is not entirely clear. It seems that the first step is an irreversible interaction of the oligosaccharide chain with the erythrocyte membrane [53,54,55,56]. Therefore, the activity of a saponin is strongly influenced by the structure of the oligosaccharide moiety. In the following step, enzymatic deglycosylation releases the aglycon, which destroys the membrane locally. From the literature [57,58], it is evident that the haemolytic activity varies considerably with the structure of the glycoside.

### 4.7. Antimicrobial Activity

The crude ethanolic extract of *GS* leaves showed significant antibacterial activity against *Bacillus pumilis*, *B. subtilis*, *Pseudomonas aeruginosa* and *Staphylococcus aureus*, but no activity against *Proteus vulgaris* or *Escherichia coli* [59]. The aqueous and methanolic extracts of the leaves also showed moderate activity against three pathogenic *Salmonella* species (*Salmonella typhi*, *S. typhimurium* and *S. paratyphi*) [60]. The chloroform and ethyl acetate extracts of the aerial parts of *GS* also exhibited activity against *P. vulgaris*, *E. coli*, *P. aeruginosa*, *Klebsella pneumoniae* and *S. aureus* [61].

### 4.8. Anti-Inflammatory Activity

The aqueous extract of *GS* leaves was investigated for anti-inflammatory activity in rats using carrageenan-induced paw oedema and the cotton pellet method at doses of 200, 300 and 500 mg/kg. The 300 mg/kg dose decreased paw oedema volume by 48.5% within 4 hours after administration [62], compared to the standard drug phenybutazone (57.6%). Additionally, doses of 200 and 300 mg/kg significantly reduced granuloma weight compared to the control group [18,62]. By elevating liver enzymes, such as γ-glutamyl transpeptidase and superoxide dismutase, extracts of *GS* show a protective effect against the release of slow-reacting substances and free radicals. The extracts did not inhibit granuloma formation or related biochemical indices, such as the hydroxyproline and collagen levels. Extracts of *GS* exhibited reduced gastrotoxicity even at high doses and did not affect the integrity of the gastric mucosa compared to other non-steroidal anti-inflammatory agents [63].

### 4.9. Anti-Larvicidal Activity

One ecological role of secondary metabolites is defence against phytophagous insects and pathogens. This suggests interesting applications in the search for new environmentally friendly, biodegradable, bioactive natural products that may avoid some of the deleterious environmental effects of synthetic pesticides [64,65,66]. Triterpenes and sterols have important roles in the acquisition of cholesterol by insects, which rely exclusively on exogenous cholesterol sources for normal growth, development, and reproduction [67]. Many terpenoids exhibit insecticidal and insect growth regulation (IGR) activities, inhibit enzymes and metabolism [68,69], and have antifeedant effects on phytophagous insects [70]. For example, aqueous extracts of *GS* show significant larvicidal activity against *Culex* larvae (44%–89% mortality in *Culex quinquefasciatus*) [71]. The extracts may also possess activity against the larvae of *Anopheles subpictus* [72].

### 4.10. Antioxidant Activity

*GS* extracts rich in oleane saponins have been examined for antioxidant activity. The IC_50_ values for DPPH scavenging, superoxide radical scavenging, inhibition of *in vitro* lipid peroxidation, and protein carbonyl formation were 238, 140, 99, and 28 μg/mL, respectively [73]. The antioxidant activity shown by the 55% v/v alcoholic *GS* extract may be due to the presence of flavonoids, phenols, tannins and triterpenoids, all of which were detected in preliminary phytochemical screening [4]. *In vivo* studies have shown that pre-treatment with *GS* extracts significantly enhanced the radiation (8 Gy)-induced augmentation of lipid peroxidation and depletion of glutathione and protein in mouse brain. Some multi-herbal ayurvedic formulations containing extracts of *GS*, such as Hyponidd and Dihar, have shown antioxidant activity by increasing the levels of superoxide dismutase, glutathione and catalase in rats [74,75].

### 4.11. Side-Effects

*GS* is considered safe when taken at the recommended doses. Short term use of low doses may have no noticeable side effects [76]. Extremely high doses may induce hypoglycaemia. Symptoms such as weakness, confusion, fatigue, shakiness, excessive sweating and loss of muscle control may occur. Gymnema may cause gastrointestinal distress, including abdominal cramping, nausea and vomiting, when taken on an empty stomach. Spontaneously hypertensive rats consuming *GS* showed no change in systolic blood pressure [45].

### 4.12. Toxicity

Several reports suggest that a reliably toxic dose of *GS* has not been found. The LD_50_ in mice and rats is greater than 5 g/kg [35]. Peroral administration of a hydro-alcoholic extract of *GS* (19.5:1) to mice at a dose of 0.25–8 g/kg did not produce any behavioural or neurological effects. Feeding studies lasting 52 weeks in rats including administration of 1% *GS* powder in the diet, showed no toxic effects; none of the animals died during this period [4]. No side effects were found upon administration of *GS* at doses of 0.50–0.56 g/kg/day in man [77]. *GS* has been reported to cause toxic hepatitis or drug-induced liver injury in patients who have been treated with this herb for diabetes mellitus [75]. D-400 (a formulation of herbs known for their hypoglycaemic actions that contains *GS* as one of the major components) showed no adverse effects on rats and no teratogenicity [78]. The plant increased the effectiveness of diabetic medication.

## 5. Discussion of the Specific Biological Activity of Tested Molecules

The aqueous extract of *GS* leaves shows potent anti-inflammatory activity in rats [62] and significant larvicidal activity, for example, against *Culex quinquefasciatus* [71] and *Anopheles subpictus* larvae [72].

Studies on the crude ethanolic extract of *GS* leaves showed significant antibacterial and anti-cancer activities. The LD_50_ values of the ethanolic and water extracts of *GS* administered intraperitoneally in mice was found to be 375 mg/kg [79]. In an acute toxicity study in mice, no gross behavioural, neurological, or autonomic effects were observed.

Not surprisingly, this plant has been and is still being studied by many research groups, as demonstrated by the publication of thousands of articles and the isolation of one hundred new and known molecules. However, few of the oleane triterpenoids isolated from *GS* that are gem-dimethylated at C-4 (see Figure 1 and Table 3 and Table 4 [80,81,82,83,84,85,86,87,88,89,90,91,92,93,94,95], Table 5 [89,95,96,97,98,99,100,101,102,103,104,105], Table 6 [81,82,83,88,89,91,92,93,94,95,103], Table 7 [51,89,95,99,100,104,105,106,107,108,109]) have been assayed (see Figure 1 and Table 8 [81,82,88,89,91,92,93,94,95,103,108,110,111,112,113,114,115] and Table 9 [51,89,95,99,102,103,104,105,108,111,112,115,116,117,118,119]).

For example, longispinogenin (**1**) was evaluated for anti-inflammatory activity in 12-*O*-tetra-decanoylphorbol-13-acetate (TPA)-induced inflammation in mice. The inhibitory effect was compared with that of a reference compound, quercetin (a known inhibitor of TPA-induced inflammation in mice), and two commercially available anti-inflammatory drugs, indomethacin and hydrocortisone. Longispinogenin (**1**) showed marked inhibitory activity, with an ID_50_ of 0.03–1.0 mg/ear, which was more potent than quercetin (1.6 mg/ear) and comparable to indomethacin (0.3 mg/ear) [113]. It also showed antitubercular activity against *Mycobacterium tuberculosis* H37Rv in Middlebrook 7H12 medium using the Microplate Alamar Blue Assay. Longispinogenin showed potent activity, although it was an order of magnitude less potent than the first-line antitubercular drug, rifampin [110]. Longispinogenin (**1**) and oleanolic acid (**17**) have been reported to strongly inhibit HIV-1 replication in H9 lymphocyte cells and in CEM 4 and MT-4 cells. However, the results of cell-free enzymatic assays should be cautiously extrapolated to the mode of action of these compounds in intact cells. In addition, these compounds were tested for inhibition of HIV-1 reverse transcriptase (RT). RT is a key enzyme for human immunodeficiency virus (HIV), as it catalyses the RNA- and DNA-dependent synthesis of double-stranded proviral DNA. Because replication of HIV is interrupted by RT inhibitors, inhibition of HIV RT is considered a useful approach for the prophylaxis of acquired immunodeficiency syndrome (AIDS). Several nucleosides; natural flavonoids, tannins and alkaloids; synthetic benzodiazepines and piperazine derivatives; and non-nucleoside-type compounds with diverse molecular structures have been reported as being HIV RT inhibitors. However, the efficacy of both nucleoside and non-nucleoside RT inhibitors is limited by high rates of viral mutation, which rapidly leads to the emergence of drug-resistant viral strains. Compounds possessing potent anti-HIV activity with novel structures and modes of action are urgently needed. Whereas oleanolic acid (**17**; 3.1 μM) was shown to possess potent inhibitory effect against HIV-1 RT, longispinogenin showed no or a weak inhibitory effect [112].

Chichipegenin (**2**) was evaluated for inhibition of the growth and development of the fall armyworm (FAW, *Spodoptera frugiperda*) and yellow mealworm (*Tenebrio molitor* L.) by examining different aspects including insecticidal and growth-regulatory activities, rate of development, time of pupation, adult emergence, and deformities at each life stage [114].

Of those reported in Table 3, only compounds **9**–**12**, **14** and **16** have been assayed for anti-sweetness activity. Solutions of these compounds (5 mL, 1 mM) were evaluated in adult volunteers using the method of Maeda *et al*. [120]. Each participant held the solution in the mouth with distilled water. Immediately after this, different concentrations of sucrose (0.5–0.1 M) were administered. The sweetness-inhibiting activity of each compound was expressed as the maximum concentration of sucrose for which sweetness was completely suppressed.

Compound **11** and the sodium salt of alternoside II (**16**) completely suppressed the sensation of sweetness. Compound **9**, alternoside VII (**10**), alternoside XIX (**14**), and compound **12**, which does not have an acyl group, were inactive even when tested at a lower concentration of sucrose (0.1 M) [92,93,94]. This finding seems to indicate that the anti-sweetness activity of these triterpene saponins is related to the presence of acyl groups on the D and E rings. This is consistent with the hypothesis that ester groups can play an important role in anti-sweetness activity.

**Table 3:** Structures of gem-dimethylated oleanes isolated from *Gymnema sylvestre*.

No.	CAS	Common Name	R_1_	R_2_	R_3_	R_4_	R_5_	R_6_
**1**	465-94-1	Longispinogenin	H	OH	CH_2_OH	H	H	CH_3_
**2**	474-15-7	Chichipegenin	H	OH	CH_2_OH	OH	H	CH_3_
**3**	53187-93-2	Sitakisogenin	H	OH	CH_2_OH	H	OH	CH_3_
**4**	862377-55-7	3β,16β,22β,28-tetrahydroxy-olean-12-en-30-oic acid	See Figure 2					
**5**	287390-11-8	Longispinogenin 3-*O*-β-d-glucuronopyranoside	**GlcA**	OH	CH_2_OH	H	H	CH_3_
**6**	287389-94-0	21β-*O*-Benzoylsitakisogenin 3-*O*-β-d-glucuronopyranoside	**GlcA**	OH	CH_2_OH	H	**Bz**	CH_3_
**7**	873799-50-9	Gymnemic acid A	**GlcA**	OH	CH_2_OH	H	H	COOH
**8**	1096581-47-3	Gymnemoside W2	**GlcA-OMe**	OH	CH_2_OH	H	OH	CH_3_
**9**	330595-34-1	Longispinogenin 3-*O*-β-d-glucopyranosyl-(1→3)-β-d-glucuronopyranoside potassium salt	**B**	OH	CH_2_OH	H	H	CH_3_
**10**	212775-47-8	Alternoside VII	**B**	OH	CH_2_OH	OH	H	CH_3_
**11**	330595-32-9	21β-*O*-Benzoylsitakisogenin 3-*O*-β-d-glucopyranosyl-(1→3)-β-d-glucuronopyranoside	**B**	OH	CH_2_OH	H	**Bz**	CH_3_
**12**	330595-36-3	29-Hydroxylongispinogenin 3-*O*-β-d-glucopyranosyl-(1→3)-β-d-glucuronopyranoside potassium salt	**B**	OH	CH_2_OH	H	H	CH_2_OH
**13**	1096581-44-0	Gymnemoside W1	**A**	OH	CH_2_O**Glc**	H	H	CH_3_
**14**	256510-01-7	Alternoside XIX	**C**	OH	CH_2_O**Glc**	H	H	CH_3_
**15**	1422031-89-7	6-Deoxy-α-l-Rhamnopyranoside, (3β,16β,22α)-16-(hydroxy)-28-[(6-deoxy-α-L-mannopyranosyl)oxy]-22-hydroxyolean-12-en-3-yl 3-*O*-β-d-glucopyranosyl	**D**	OH	CH_2_O**(6-Deoxy-α-l)Man**	OH	H	CH_3_
**16**	212775-23-0	Alternoside II	**B**	OAc	CH_2_O**(6-Deoxy-α-l)Man**	OH	H	CH_3_
**17**	508-02-1	Oleanolic acid	H	H	H	H	H	CH_3_
**18**	240140-86-7	Oleanolic acid 3-*O*-β-d-glucopyranosyl-(1→6)-β-d-glucopyranoside	**A**	H	COOH	H	H	CH_3_
**19**	287389-96-2	Oleanolic acid 3-*O*-β-d-xylopyranosyl(1→6)-β-d-glucopyranosyl(1→6)-β-d-glucopyranoside	**C**	H	COOH	H	H	CH_3_
**20**	14162-53-9	Oleanolic acid 28-*O*-β-d-glucopyranoside	H	H	COO**Glc**	H	H	CH_3_
**21**	78454-20-3	Silphioside B	**Glc**	H	COO**Glc**	H	H	CH_3_
**22**	287389-95-1	3-*O*-β-d-Glucopyranosyl(1→6)-β-d-glucopyranosyl oleanolic acid 28-*O*-β-d-glucopyranosyl ester	**A**	H	COO**Glc**	H	H	CH_3_
**23**	287389-97-3	3-*O*-β-d-Xylopyranosyl(1→6)-β-d-glucopyranosyl(1→6)-β-d-glucopyranosyl oleanolic acid 28-β-d-glucopyranosyl ester	**C**	H	COO**Glc**	H	H	CH_3_
**24**	287389-98-4	3-*O*-β-d-Glucopyranosyl(1→6)-β-d-glucopyranosyl oleanolic acid 28-β-d-glucopyranosyl(1→6)-β-d-glucopyranosyl ester	**A**	H	COO**-A**	H	H	CH_3_
**25**	1422031-87-5	3β,16β,22α-Trihydroxy-olean-12-ene 3-*O*-β-d-xylopyranosyl-(1→6)-β-d-glucopyranosyl-(1→6)-β-d-glucopyranoside	**C**	OH	CH_3_	OH	H	CH_3_

**Table 4:** Purification and biophysical properties of oleanes gem-dimethylated isolated from *Gymnema sylvestre*.

No.	Chromatographic conditions	Melting point (°C)	IR analysis (cm^−1^)	Mass analysis	[α]_D_ (Concentration, Solvent)	Reference
**1**	-	247–249	-	-	+53 (Acetone)	[80]
	Preparative TLC (CH_2_Cl_2_/Me_2_CO, 17:3)	-	-	-	-	[81]
	-	216–218	-	EI: [M]^+^ 458	+38.7 (c2.5, CHCl_3_)	[82]
				HR-ESI: [M]^+^ 458.3755		
	-	218–220	-	-	+51.0 (CHCl_3_)	[83]
	-	244–245	-	-	-	[84]
**2**	By synthesis	315–317	-	HR-ESI: [M-H_2_O]^+^ 456.3580	+35.4 (c1.2, CHCl_3_)	[85]
	-	321–323	-	-	+43 (c1, CHCl_3_)	[86]
	HPLC (RP-C18; CH_3_OH/CH_3_CN/H_2_O, 2:7:1)	-	-	-	-	[81]
**3**	HPLC (RP-C18; CH_3_OH/CH_3_CN/H_2_O, 2:5:3)	-	-	-	-	[81]
	HPLC (S-5; H_2_O/CH_3_CN, 63:37)	333–335	-	HR-ESI: [M-H_2_O]^+^ 456.3628	+57.0 (c0.9, CHCl_3_:CH_3_OH 1:1)	[82,87]
**4**	-	-	-	ESI: [M+H]^+^ 505, [M+Na]^+^ 527	-	[88]
**5**	Silica gel column chromatography using as eluent CHCl_3_/CH_3_OH	198–202	3414 (OH), 1724	FAB: [M+Na]^+^ 657	+16.08 (c0.10, CH_3_OH).	[89]
			(COOH), 1636 (C=C), 1458, 1380, 1054.			
**6**	Silica gel column chromatography using as eluent CHCl_3_/CH_3_OH	192–195	3444 (OH), 1724, 1700, 1635 (C=C), 1457, 1388, 1280, 1074, 720.	FAB: [M+Na]^+^ 777	+27.2 (c0.15, CH_3_OH).	[89]
**7**	-	-	-	-	-	[90]
**8**	HPLC (RP-C18; CH_3_OH/H_2_O, 4:1) and Si-60 (CHCl_3_/CH_3_OH, 10:1 to 5:2)	-	-	HR-ESI: [M+Na]^+^ 687.4085	−6.5 (c0.01, CH_3_OH)	[91]
**9**	HPLC (RP-C18; CH_3_OH/H_2_O, 1:4 to 7:3)	305–310	3440, 2948, 1636, 1420, 1078, 1028	HR-ESI: [(M^.^K)+H]^+^ 835.4065	+18.1 (c0.08, CH_3_OH)	[92]
**10**	HPLC (ODS S-5, H_2_O/CH_3_CN, 4:1)	213–215	3460, 1720, 1655, 1155	FAB: [M−H]^−^ 811	+9.1 (c4.5, CH_3_OH)	[93]
**11**	HPLC (RP-C18; CH_3_OH/H_2_O, 1:4 to 7:3)	226–228	3422, 2948, 1702, 1636, 1460, 1388, 1282, 1160, 1076, 1026	FAB: [M+H]^+^ 917, [M+Na]^+^ 939	+15.4 (c0.16, CH_3_OH)	[92]
**12**	HPLC (RP-C18; CH_3_OH/H_2_O, 1:4 to 7:3)	290–293	3422, 2928, 1618, 1430, 1028	FAB: [(M^.^K)+H]^+^ 851	+10.3 (c0.12, CH_3_OH)	[92]
**13**	HPLC (RP-C18; CH_3_OH/H_2_O, 4:1) and Si-60 (CHCl_3_/CH_3_OH, 10:1 to 5:2)	-	-	ESI: [M-H]^−^ 943	−26.6 (c0.004, CH_3_OH)	[91]
				[M-Glc]^−^ 781, [M-2×Glc]^−^ 619		
				[M-3×Glc]^−^ 457, [M+Na]^+^ 967		
				HR-ESI: [M]^+^ 944.5290		
**14**	HPLC (YMC; S-5; H_2_O/CH_3_CN 3:1 to 13:7)	187–189	3450, 1155	FAB: [M-H]^−^ 1075	−23.4 (c3.1, CH_3_OH)	[94]
**15**	-	-	-	-	-	[95]
**16**	HPLC (RP-C18; CH_3_OH/H_2_O, 1:4 to 7:3)	294–296	3418, 2948, 1738, 1713, 1621, 1430, 1374, 1266, 1076, 1031	FAB: [(M^.^K)+H]^+^ 1023	+1.5 (c0.19, CH_3_OH)	[92]
	HPLC (ODS S-5; H_2_O/CH_3_CN, 4:1)	230–232	3400, 1730, 1665, 1240, 1160	FAB: [M-H]^−^ 999	+2.3 (2.4, CH_3_OH)	[93]

**Table 5:** Purification and biophysical properties of oleanes gem-dimethylated isolated from *Gymnema sylvestre*.

No.	Chromatographic conditions	Melting point (°C)	IR analysis (cm^−1^)	Mass analysis	[α]_D _(Concentration, Solvent)	Reference
**17**	HPLC (RP-C18; CH_3_OH/H_2_0, 30:1)	296–298	3420, 2930, 1680	EI: 456, 438, 248, 207, 203, 189	+70.0 (c0.4, CHCl_3_)	[96]
	HPLC (RP-C18; CH_3_OH/H_2_0, 4:1)	-	-	ESI: [M+CH_3_OH+ Na]^+^ 511, [M+Na]^+^ 479, 203, 191.	-	[97]
	HPLC (RP-C18; CH_3_OH/Acetic acid/Triethylamine, 99.55:0.30:0.15)	-	-	ESI: [M−H]^−^ 455	-	[98]
**18**	Preparative TLC (CH_2_Cl_2_/CH_3_OH, 4:1)	220 (decomp.)	3375, 2943, 1559, 1459, 1385, 1311, 1074, 1032, 913, 630	LSI (%): [M^+^−H] 779 (18.4), 777(2.5), 733(0.5), 645(0.6), 617(3.0), 551(0.8), 497(0.5), 483(1.3), 455(2.8), 437(1.3), 367(6.0), 331(0.8), 275(24.2), 273(5.3), 183(100.0), 151(6.0), 91(100.0), 71(12.1), 45 (1.5)	+4.2 (c0.24, CH_3_OH)	[99]
**19**	HPLC (RP-C18; CH_3_OH/H_2_O, 3:7 to 7:3)	202–204	3410 (OH), 1710 (COOH), 1638 (C=C), 1458, 1036.	FAB: [M+Na]^+^ 935	−3.28 (c0.15, CH_3_OH)	[89]
**20**	Silica gel column chromatography using as eluent CHCl_3_/CH_3_OH/H_2_O, 10:3:1 (lower layer)	-	-	ESI: [M+Na]^+^ 641.2	-	[100,101]
	CC silica gel, using EtOAc					[102]
	Flash chromatography (CH_2_Cl_2_/CH_3_OH, 20:1)	224–226	3435, 2944, 2873, 1735, 1460, 1385, 1072, 1029	ESI: [M+Na]^+^ 641.4	-	[103]
**21**	HPLC (RP-C18; CH_3_OH/H_2_O, 6:4)	-	-	FAB: [M−H]^−^ 779,	-	[104]
				[(M−H)−162]^−^ 617,		
				[(M−H)−178]^−^ 601		
	HPLC (RP-C8; CH_3_OH/H_2_O)	-	-	HR-ESI: [M+Na]^+^ 803.4509	-	[105]
	HPLC (RP-C18; CH_3_OH/H_2_O, 13:7)	230	-	Incorrect data	−6.3 (c0.17, MeOH)	[103]
**22**	HPLC (RP-C18; CH_3_OH/H_2_O, 3:7 to 7:3)	206–209	3424 (OH), 1735 (COOH), 1636 (C=C), 1457, 1034	FAB: [M+Na]^+^ 943	−6.5 (0.11, MeOH)	[89]
**23**	HPLC (RP-C18; CH_3_OH/H_2_O, 3:7 to 7:3)	212–215	3414 (OH), 1740 (COOR), 1636 (C=C), 1460, 1364, 1044, 896	FAB: [M+Na]^+^ 1097	−9.68 (c0.20, MeOH).	[89]
**24**	HPLC (RP-C18; CH_3_OH/H_2_O, 3:7 to 7:3)	209–211	3424 (OH), 1734 (COOR), 1636 (C=C), 1458, 1074.	FAB: [M+Na]^+^ 1127	−12.18 (c0.12, MeOH)	[89]
**25**	-	-	-	-	-	[95]

**Table 6:** Systematic names and chemical and spectral data of compounds **1**–**16**.

No.	Mol. formula	Mol. weight	Aspect	^1^H-NMR	^13^C-NMR	Systematic Name	Reference
**1**	C_30_H_50_O_3_	458.72	Amorphous powder	CDCl_3_	CDCl_3_	Olean-12-ene-3β,16β,28-triol	[81,82,83]
				-	C_5_D_5_N		[93]
**2**	C_30_H_50_O_4_	474.72	Amorphous powder	C_5_D_5_N	C_5_D_5_N	Olean-12-ene-3β,16β,22α,28-tetrol	[81,82]
**3**	C_30_H_50_O_4_	474.72	Amorphous powder	C_5_D_5_N	C_5_D_5_N	Olean-12-ene-3β,16β,21β,28-tetrol	[81,83]
**4**	C_30_H_48_O_6_	504.70	White amorphous powder	C_5_D_5_N	C_5_D_5_N	Olean-12-en-30-oic acid, 3,16,22,28-tetrahydroxy-, (3β,16β,20β,22β) ^a^	[88]
**5**	C_36_H_58_O_9_	834.84	Amorphous powder	C_5_D_5_N	C_5_D_5_N	β-d-Glucopyranosiduronic acid, (3β,16β)-16,28-dihydroxyolean-12-en-3-yl	[89]
**6**	C_43_H_62_O_11_	754.95	Amorphous powder	C_5_D_5_N	C_5_D_5_N	β-d-Glucopyranosiduronic acid, (3β,16β,21β)-21-benzoyloxy-16,28-dihydroxyolean-12-en-3-yl	[89]
**7**	C_36_H_56_O_11_	664.82	N. a.	N. a.	N. a.	β-d-Glucopyranosiduronic acid, (3β,16β,20α)-20-carboxy-16,28-dihydroxy-30-norolean-12-en-3-yl	[103]
**8**	C_37_H_60_O_10_	664.87	White powder	C_5_D_5_N	C_5_D_5_N	β-d-Glucopyranosiduronic acid, (3β,16β,21β)-16,21,28-trihydroxyolean-12-en-3-yl, methyl ester	[91]
**9**	C_42_H_68_O_14_K	836.08	Amorphous powder	C_5_D_5_N	C_5_D_5_N	β-d-Glucopyranosiduronic acid, (3β,16β)-16,28-dihydroxyolean-12-en-3-yl 3-*O*-β-d-glucopyranosyl-, monopotassium salt	[92]
**10**	C_42_H_68_O_15_	812.98	Colorless needles	C_5_D_5_N	C_5_D_5_N	β-d-Glucopyranosiduronic acid, (3β,16β,22α)-16,22,28-trihydroxyolean-12-en-3-yl 3-*O*-β-d-glucopyranosyl	[93]
**11**	C_49_H_72_O_16_	917.09	Amorphous powder	C_5_D_5_N	C_5_D_5_N	β-d-Glucopyranosiduronic acid, (3β,16β,21β)-21-benzoyloxy-16,28-dihydroxyolean-12-en-3-yl 3-*O*-β-d-glucopyranosyl	[92]
**12**	C_42_H_68_O_15_K	852.08	Amorphous powder	C_5_D_5_N	C_5_D_5_N	β-d-Glucopyranosiduronic acid, (3β,16β,20α)-16,28,29-trihydroxyolean-12-en-3-yl 3-*O*-β-d-glucopyranosyl-, monopotassium salt	[92]
**13**	C_48_H_80_O_18_	945.14	White powder	C_5_D_5_N	C_5_D_5_N	β-d-Glucopyranoside, (3β,16β)-28-β-d-glucopyranosyloxy-16-hydroxyolean-12-en-3-yl 6-*O*-β-d-glucopyranosyl	[91]
**14**	C_53_H_88_O_22_	1077.25	Colorless needles	C_5_D_5_N	C_5_D_5_N	β-d-Glucopyranoside, (3β,16β)-28-β-d-glucopyranosyloxy-16-hydroxyolean-12-en-3-yl 6-[β-d-xylopyranosyl-(1→6)-*O*-β-d-glucopyranosyl]	[94]
**15**	C_48_H_80_O_17_	929.14	White amorphous powder	N. a.	N. a.	6-Deoxy-α-l-Rhamnopyranoside, (3β,16β,22α)-16-(hydroxy)-28-[(6-deoxy-α-L-mannopyranosyl)oxy]-22-hydroxyolean-12-en-3-yl 3-*O*-β-d-glucopyranosyl	[95]
**16**	C_50_H_80_O_20_	1001.16	Colorless needles	C_5_D_5_N	C_5_D_5_N	β-d-Glucopyranosiduronic acid, (3β,16β,22α)-16-acetyloxy-28-[(6-deoxy-α-L-mannopyranosyl)oxy]-22-hydroxyolean-12-en-3-yl 3-*O*-β-d-glucopyranosyl	[92,93]

^a^ There is no correspondence between the structure and name of the compound reported in the literature and as indicated by the CAS number; N. a. = Not available.

**Table 7:** Systematic names and chemical and spectral data of compounds **17**–**25**.

No.	Mol. formula	Mol. weight	Aspect	^1^H-NMR	^13^C-NMR	Systematic Name	Reference
**17**	C_30_H_48_O_3_	456.70	White solid	C_5_D_5_N	C_5_D_5_N	3β-Hydroxyolean-12-en-28-oic acid	[107]
				CD_3_OD	CD_3_OD		[106]
**18**	C_42_H_68_O_13_	780.98	White solid	CD_3_OD	CD_3_OD	Olean-12-en-28-oic acid, 3-[(6-*O*-β-d-glucopyranosyl-β-d-glucopyranosyl)oxy]-, (3β)	[99]
				C_5_D_5_N	C_5_D_5_N		[51]
**19**	C_47_H_76_O_17_	913.10	Amorphous powder	C_5_D_5_N	C_5_D_5_N	Olean-12-en-28-oic acid, 3-[(*O*-β-d-xylopyranosyl-(1→6)-*O*-β-d-glucopyranosyl-(1→6)-β-d-glucopyranosyl)oxy]-, (3β)	[89]
**20**	C_36_H_58_O_8_	618.84	Colorless powder	C_5_D_5_N	C_5_D_5_N	Oleanolic acid 28-*O*-β-d-glucopyranoside	[100]
**21**	C_42_H_68_O_13_	780.98	White amorphous powder	-	CDCl_3_	3-*O*-(β-d-Glucopyranosyl)-oleanolic acid-28-*O*-β-d-glucopyranoside	[108]
				-	C_5_D_5_N		[105]
				CD_3_OD	CD_3_OD		[104]
**22**	C_48_H_78_O_18_	943.12	Amorphous powder	C_5_D_5_N	C_5_D_5_N	Olean-12-en-28-oic acid, 3-[(6-*O*-β-d-glucopyranosyl-β-d-glucopyranosyl)oxy]-, β-d-glucopyranosyl ester, (3β)-	[89,109]
**23**	C_53_H_86_O_22_	1075.24	Amorphous powder	C_5_D_5_N	C_5_D_5_N	Olean-12-en-28-oic acid, 3-[(*O*-β-d-xylopyranosyl-(1→6)-*O*-β-d-glucopyranosyl-(1→6)-β-d-glucopyranosyl)oxy]-, β-d-glucopyranosyl ester, (3β)	[89,95]
**24**	C_54_H_88_O_23_	1105.26	Amorphous powder	C_5_D_5_N	C_5_D_5_N	Olean-12-en-28-oic acid, 3-[(6-*O*-β-d-glucopyranosyl-β-d-glucopyranosyl)oxy]-, 6-*O*-β-d-glucopyranosyl-β-d-glucopyranosyl ester, (3β)	[89]
**25**	C_47_H_78_O_17_	915.11	N. a.	N. a.	N. a.	3β,16β,22α-Trihydroxy-olean-12-ene 3-*O*-β-d-xylopyranosyl-(1→6)-β-d-glucopyranosyl-(1→6)-β-d-glucopyranoside	[95]

N. a. = Not available.

**Table 8:** Biological activity of compounds **1**–**16** and their natural sources.

No.	Part of the plant	Extract	Reference	Activity	Reference
**1**	Stems	EtOH	[110]	Antitubercular activity against *Mycobacterium tuberculosis *strain H37Rv	[110]
	Leaves	EtOH/H_2_O (19:1)	[111]	Inhibition on human immunodeficiency virus type 1 (HIV-1) reverse transcriptase	[112]
	Aerial parts	CH_2_Cl_2_	[81]	Anti-inflammatory	[113]
**2**	Aerial parts	CH_2_Cl_2_	[81]	Insect growth regulatory against *Spodoptera frugiperda* and *Tenebrio molitor*	[114]
	Leaves	EtOH/H_2_O (19:1)	[111]		
	Stems	EtOH	[110]		
	Entire part	MeOH/CH_2_Cl_2_ (1:1)	[114]		
**3**	Aerial parts	CH_2_Cl_2_	[81]	-	-
	Leaves	EtOH/H_2_O (19:1)	[111]		
	Leaves	EtOH	[108]		
	Stems	N. a.	[82]		
**4**	Leaves	Not specified	[88]	-	-
**5**	Leaves	EtOH	[89]	Prevention or treatment of disorders related to high blood sugar, high blood lipids, or blood clotting	[115]
**6**	Leaves	EtOH	[89]	Prevention or treatment of disorders related to high blood sugar, high blood lipids, or blood	[115]
**7**	N. a.	N. a.	[103]	-	-
**8**	Leaves	EtOH/H_2_O (4:1)	[91]	-	-
**9**	Leaves	EtOH/H_2_O (3:2)	[92]	Antisweet activity	[92]
**10**	Roots	EtOH/H_2_O (7:3)	[93]	Antisweet activity	[93]
**11**	Leaves	EtOH/H_2_O (3:2)	[92]	Antisweet activity	[92]
**12**	Leaves	EtOH/H_2_O (3:2)	[92]	Antisweet activity	[92]
**13**	Leaves	EtOH/H_2_O (4:1)	[91]	-	-
**14**	Roots	EtOH	[94]	Antisweet activity	[94]
**15**	Stems	N. a.	[95]	-	-
**16**	Roots	EtOH/H_2_O (7:3)	[93]	Antisweet activity	[93]
	Leaves	EtOH/H_2_O (3:2)	[92]	Antisweet activity	[92]

**Table 9:** Biological activity of compounds **17**–**25** and their natural sources.

No.	Part of the plant	Extract	Reference	Activity	Reference
**17**	Leaves Leaves	EtOH/H_2_O (19:1) H_2_O+microwave	[111] [116,117]	Inhibition on human immunodeficiency virus type 1 (HIV-1) reverse transcriptase	[112]
				Glucose uptake and gastric emptying	[101,118]
				Inhibition of Glycogen Phosphorylase	[108]
**18**	Stems	N. a.	[95]	Haemolytic Activity	[99]
	Obtained by synthesis		[99]		
**19**	Leaves	EtOH	[89]	Prevention or treatment of disorders related to high blood sugar, high blood lipids, or blood	[115]
**20**	Leaves	N. a.	[108]	Glucose uptake and gastric emptying	[101,118]
				Haemolytic Activity	[51]
				Inhibition of Glycogen Phosphorylase	[103]
				Antimicrobial Activities	[119]
**21**	Leaves	N. a.	[108]	Inhibition of the growth of HL60, A549 and	[105] [119]
	Leaves	EtOH/H_2_O (3:2)	[105]	DU145 cancer cells	
	Leaves	MeOH/CH_2_Cl_2_ (1:1)	[104]	Amylase activity, total protein content and seedling growth	
**22**	Leaves	EtOH	[89]	Prevention or treatment of disorders related to high blood sugar, high blood lipids, or blood	[115]
**23**	Leaves	EtOH	[89]	Prevention or treatment of disorders related to high blood sugar, high blood lipids, or blood	[115]
**24**	Leaves	EtOH	[89]	Prevention or treatment of disorders related to high blood sugar, high blood lipids, or blood	[115]
**25**	Stems	N. a.	[95]	-	-

N. a. = Not available

Compounds **5**, **6**, **19**, **22**−**24** are useful in the prevention or treatment of disorders related to high blood sugar and lipids or to blood clotting. In particular, compound **24** was tested in KunMing mice for inhibition of elevated blood glucose concentrations [115].

Only compound **18** has been evaluated for haemolytic activity (expressed as the Haemolytic Index (HI)) using the Austrian Pharmacopoeia method, with the Austrian Saponin standard HI.30000 as the reference [99]. Linkage of the terminal glucose to position 3 results in a compound with potent activity. Considering the significantly differing haemolytic indices for glycoside analogues, the investigation of other oleanolic acid glycosides seems promising. Furthermore, the effect of the configuration of the anomeric carbon attached to the aglycone should be investigated.

Oleane-type triterpene saponins are known to possess cytotoxic activity, but only silphioside B (**21**) was tested *in vitro* for cytotoxicity against three human tumour cell lines using the MTT assay: leukaemia (HL60), lung cancer (A549) and prostate cancer (DU145) [105]. This compound showed modest cytotoxicity. However, considering that this plant is both a medicine and a food, further studies should also be performed on other compounds. The influence of the oleanolic acid glycoside **21** on amylase activity and total protein content in wheat seedlings was studied [119]. Treatment of *Triticum aestivum* L. seeds with 1–10 μM aqueous solutions of **21** increased the α-amylase levels, total amylase activity and total protein content to an extent comparable to gibberellin A3 and 6-benzylaminopurine.

Compounds that reduce postprandial hyperglycaemia by inhibiting the absorption of carbohydrates have been shown to be effective for the prevention and the treatment of non-insulin-dependent diabetes mellitus. With this aim, oleanolic acid (**17**) and its 28-glucopyranoside derivative **20** were assayed to evaluate their effects on serum glucose in glucose-loaded and normal rats, but they showed no activity [101,118]. In addition, their anti-pruritic and gastromucosal protective effects have been evaluated in mice. Further experiments are necessary to gain insight into the mechanisms of action involved in these effects of saponins.

Compounds **17** and **20** showed good inhibition of rabbit muscle glycogen phosphorylase-a (GPa) monitored using a microplate reader (BIO-RAD) [108]. Briefly, GPa activity was measured by the release of phosphate from glucose 1-phosphate. Each test compound was diluted to different concentrations for IC_50_ determination, which was estimated by fitting the inhibition data to a concentration-effect curve using a logarithmic equation.

Pentacyclic triterpenes may exert hypoglycaemic effects, at least partially through GPa inhibition, but other mechanisms may also account for these effects. Further studies are needed to elucidate these molecular mechanisms in detail and to biologically evaluate natural and synthetic pentacyclic triterpenes as anti-diabetic agents.

Compound **20** has been tested for haemolytic activity using human erythrocytes in place of sheep cells. The corresponding haemolytic dose was defined as the final concentration that resulted in 50% of the haemolysis being caused by hypotonicity [51]. Comparing the results for oleanolic acid **17** with its 3- and 28-glucopyranoside (**20**) derivatives indicates that the sugar chain at the C-3 of oleanolic acid is essential, but that the sugar chain at C-28 results in inhibition of cytolytic activity; consequently, compound **20** exhibits little cytolytic activity.

## 6. Conclusions

Many Indian herbs are being used in traditional practice to cure diabetes. *GS* has an important place among such anti-diabetic medicinal plants and can also be used to treat dyspepsia, constipation, and jaundice, haemorrhoids, renal and vesicular calculi, cardiopathy, asthma, bronchitis, amenorrhoea, conjunctivitis and leucoderma. *GS* is a source of biologically active substances. A very broad spectrum of pharmacological activities has been reported, despite the fact that only a small proportion of isolated molecules were tested and that not all biological activities were evaluated. Some extracts and/or isolated compounds at various doses and in various combinations are beneficial in, among other things, the treatment of latent diabetes mellitus and the complex treatment of insulin-independent DM. In particular, some triterpenoids prolong the actions of hypoglycaemic preparations and promote regeneration of β-cells in insulin-dependent and insulin-independent DM. There has been rapid development in isolation and characterisation techniques as well as in *in vivo* studies using various rat and mouse models of life-threatening diseases. These compounds obviously require physicochemical characterisation, biological evaluation, toxicity studies, investigation of the molecular mechanism(s) of action and clinical trials. These classical approaches are necessary in the search for new lead molecules for the management of various diseases. Diabetes is becoming common throughout the world and many new drugs are being synthesised for treatment of this disorder. Many Indian herbs are being used in traditional practice to cure diabetes. *GS* has an important place among such anti-diabetic medicinal plants and can also be used to treat dyspepsia, constipation and jaundice, haemorrhoids, renal and vesicular calculi, cardiopathy, asthma, bronchitis, amenorrhoea, conjunctivitis and leucoderma. In future studies, compounds isolated from *Gurmar* must be evaluated scientifically using innovative experimental models and clinical trials to understand their mechanisms of action and to search for other active constituents in order for other therapeutic uses to be widely explored.
